# *Candida* infections in severe acute pancreatitis: we need to do more in order to distinguish invasive infection from simple colonization

**DOI:** 10.1186/s13054-020-02873-y

**Published:** 2020-04-16

**Authors:** Patrick M. Honore, Aude Mugisha, Luc Kugener, Sebastien Redant, Rachid Attou, Andrea Gallerani, David De Bels

**Affiliations:** ICU Department, Centre Hospitalier Universitaire Brugmann-Brugmann University Hospital, Place Van Gehuchtenplein, 4, 1020 Brussels, Belgium

We read with great interest the recent paper regarding anti-infective therapy for severe acute pancreatitis (SAP) by Montravers et al., who conclude regarding fungal infections that most of their cases received azoles in the context of both documented and empirical antifungal therapies [1]. We would like to make some comments. *Candida* is present in huge quantity in the colon. An increase over the years in *Candida* infection in non-neutropenic critically ill patients has been demonstrated to put them at increased risk of mortality and morbidity [2]. While there is a concern that this is the case in patients with SAP [3], this has not been universally demonstrated [4]. It is, however, likely that colonization plays an important instigating role in these invasive infections [4]. Patients with SAP are at a particular risk of invasive *Candida* infections. In a study by Hall and colleagues, both colonization with *Candida* spp. and a *Candida* colonization index score (CCIS) > 0.5 were associated with subsequent infection [4]. The CCIS was calculated for each patient as follows: CCIS = ratio of the number of non-blood distinct body sites colonized with *Candida* spp. to the total number of body sites cultured [4]. A CCIS ≥ 0.5 predicts *Candida* infection; therefore, patients who had invasive *Candida* infections and a CCIS ≥ 0.5 were defined as true positives [4]. In their commentary regarding the Hall paper, Montravers et al. themselves concluded that the mistakes of previous decades in the field of bacterial infection should not be repeated; a step-by-step approach is required [5]. Additional scientifically rigorous studies with accurate descriptions of cases similar to those in the article by Hall et al. [4] are required before prophylaxis or extensive therapeutic indications of antifungal agents in SAP can be proposed [5]. In conclusion, we believe that the cohort study of Montravers et al. should have discussed in more detail the importance of distinguishing between invasive *Candida* infections and colonization, noting, for instance, the utility of scoring systems such as the CCIS score in preventing unnecessary treatment for *Candida* colonization.

## Data Availability

Not applicable.

## Abbreviations

SAP: Severe acute pancreatitis
CCIS: *Candida* colonization index score
